# Supplementary material for: Low-dose penicillin in early life induces long-term changes in murine gut microbiota, brain cytokines and behavior
Source: Nat Commun. 2017 Apr 4;8:15062. doi: 10.1038/ncomms15062 (PMC5382287; doi:10.1038/ncomms15062)
Supplement: Supplementary Information — Supplementary Figures and Supplementary Tables [file ncomms15062-s1.pdf]

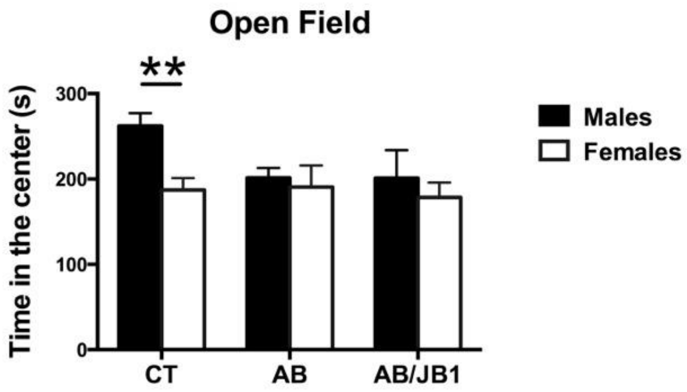

Supplementary Fig. 1: Time spent in the central area of the open field

Factorial ANOVA reveals a significant main effect of sex only. Results are means ± SEM, n = 71 (Males, n = 10 CT, 12 AB, 6 AB/JB1: Females, n = 17 CT, 13 AB, 13 AB/JB1. ** $P < 0.01$. CT: control; AB: antibiotic; AB/JB1: antibiotic and *L. rhamnosus* JB-1.

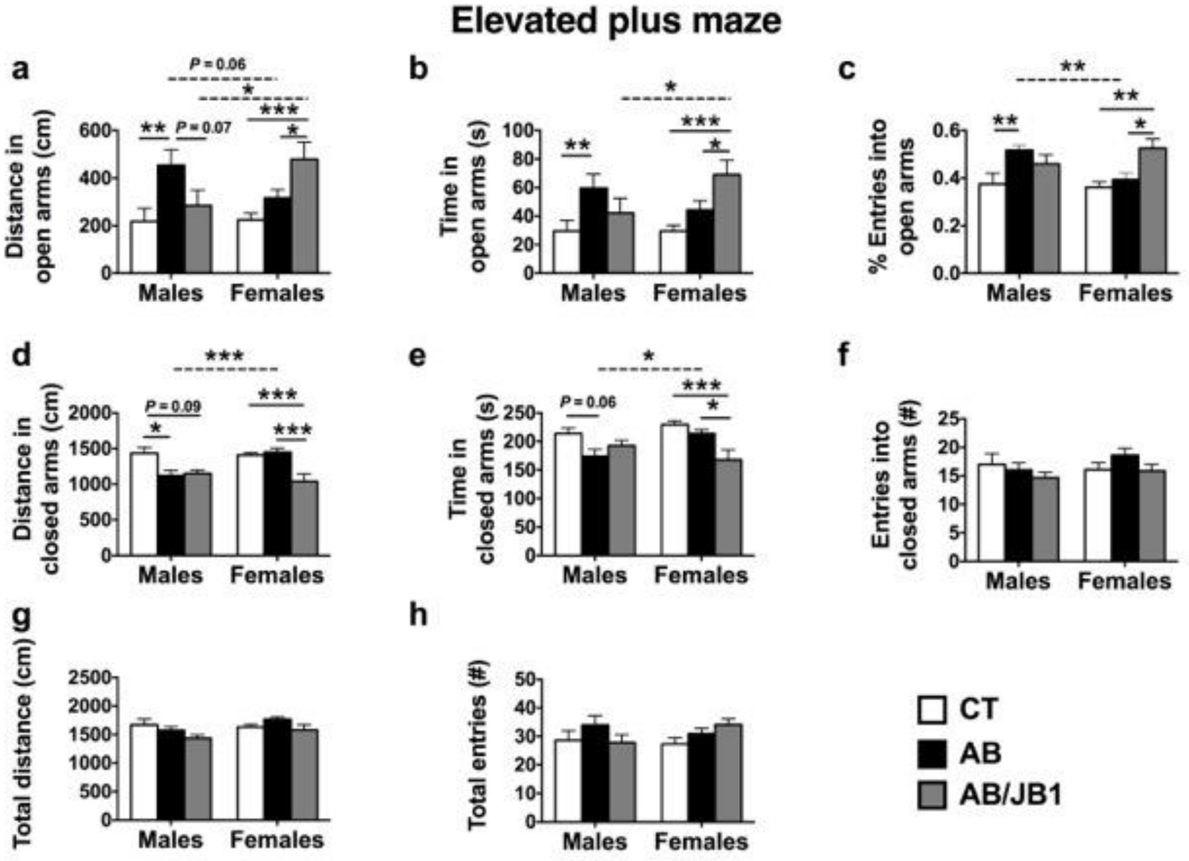

Supplementary Fig. 2: **Decreased anxiety-like behavior in males treated with AB and in females treated with AB/JB1 assessed by elevated plus maze (EPM)**

(**a-c**) Two-way ANOVA analysis revealed a main effect of treatment ($F_{2,65} = 6.175$, $P = 0.004$ / $F_{2,65} = 6.219$, $P = 0.003$ / $F_{2,65} = 7.11$, $P = 0.002$) and a significant interaction treatment*sex ($F_{2,65} = 3.19$, $P = 0.048$ / $F_{2,65} = 4.037$, $P = 0.022$ / $F_{2,65} = 3.75$, $P = 0.03$) for the time spent in the open arms, the distance travelled in the open arms and the ratio of the number of entries in the open arms to the total number of entries, respectively. No main effect of sex was observed. (**d, e**) A main effect of treatment ($F_{2,65} = 8.84$, $P < 0.001$ / $F_{2,65} = 6.28$, $P = 0.003$) and treatment*sex interaction ($F_{2,65} = 5.04$, $P = 0.009$ / $F_{2,65} = 3.38$, $P = 0.04$) were observed for distance travelled

and the time spent in the closed arms, respectively. No significant main effect of sex was observed. (**f-h**) Analysis of data reflecting locomotor activity (number of entries in the closed arms, total distance travelled in the EPM and total number of entries) show no differences between groups. When the interaction effect was significant, post-hoc tests with Bonferroni correction were performed and significant differences are reported in the graphs. Results are means ± SEM, n = 71 (Males, n = 10 CT, 12 AB, 6 AB/JB1: Females, n = 17 CT, 13 AB, 13 AB/JB1). * $P < 0.05$, ** $P < 0.01$, *** $P < 0.001$. CT: control; AB: antibiotic; AB/JB1: antibiotic and *L. rhamnosus* JB-1.

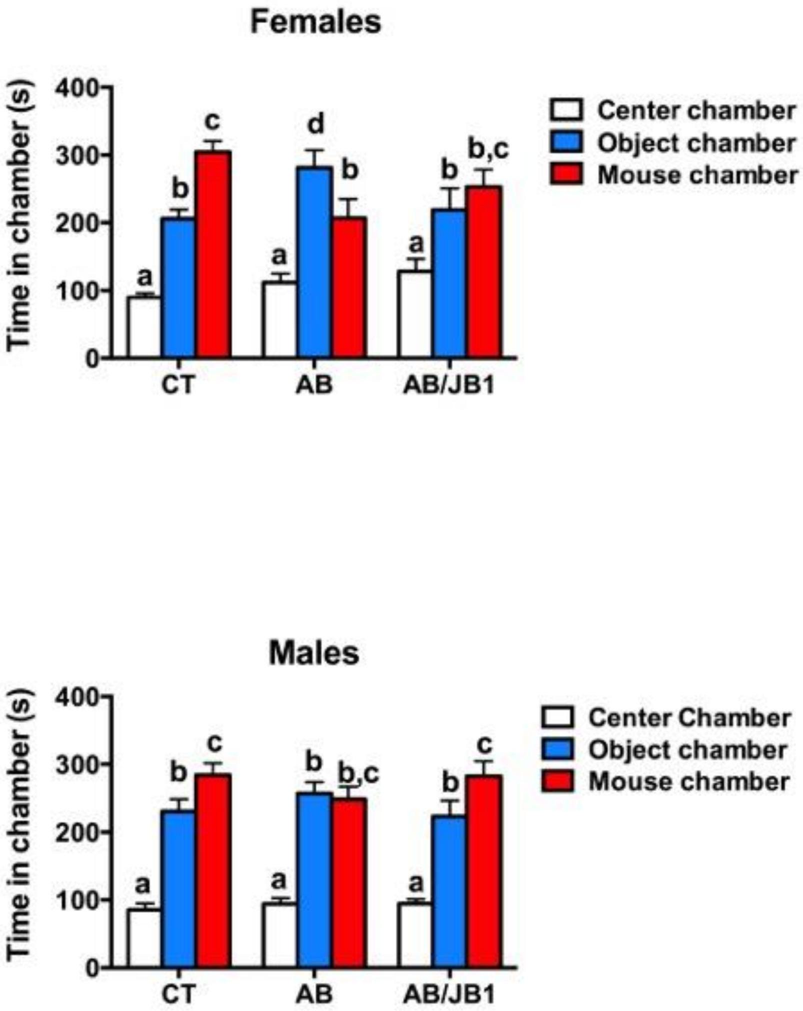

Supplementary Fig. 3: **Social behavior in females and males**

Data on time spent in the center, object and mouse chambers were analyzed with two-way ANOVA (factor 1: chamber, factor 2: treatment), followed by Tukey's multiple comparisons post-hoc tests. Results are means ± SEM (29 Males, n = 11 CT, 12 AB, 6 AB/JB1; 40 Females, n = 16 CT, 12 AB, 12 AB/JB1). Different superscript letters indicate statistically significant differences, $P < 0.05$. CT: control; AB: antibiotic; AB/JB1: antibiotic and *L. rhamnosus* JB-1.

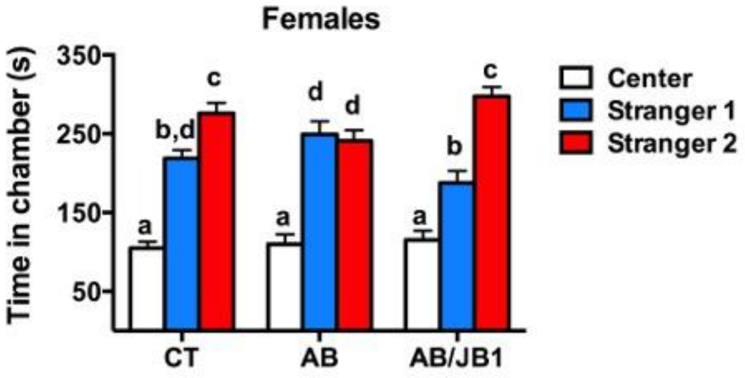

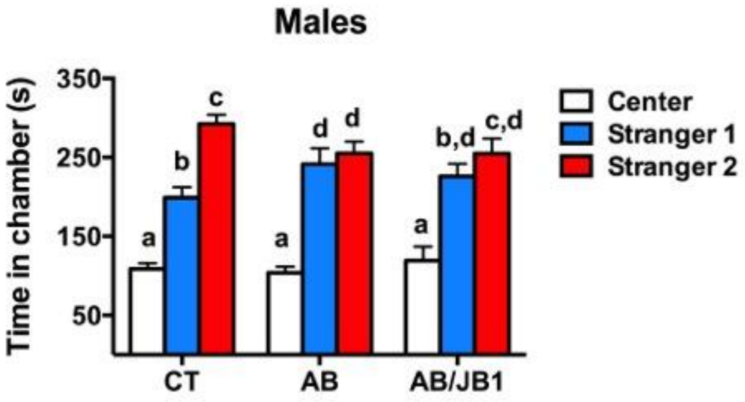

Supplementary Fig. 4: **Preference for social novelty in females and males**

Data on time spent in the center, stranger 1 and stranger 2 chambers were analyzed with two-way ANOVA (factor 1: chamber, factor 2: treatment), followed by Tukey's multiple comparisons post-hoc tests. Results are means ± SEM (29 Males, n = 11 CT, 12 AB, 6 AB/JB1; 40Females, n = 16 CT, 12 AB, 12 AB/JB1). Different superscript letters indicate statistically significant differences, $P < 0.05$. CT: control; AB: antibiotic; AB/JB1: antibiotic and *L. rhamnosus* JB-1.

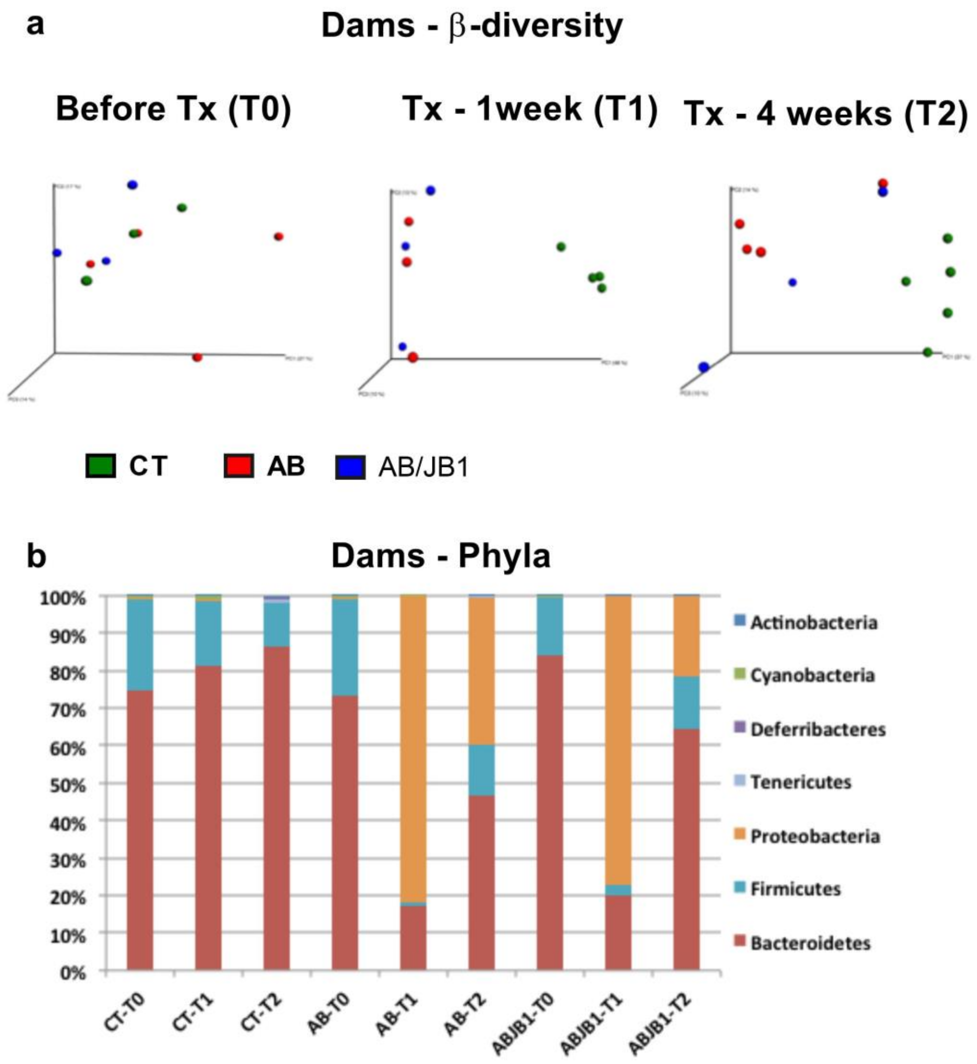

Supplementary Fig. 5: **Gut microbiota composition in dams assessed before treatment (T0) and after 1-week (T1) and 4 weeks of treatment (T2)**

(**a**) β-diversity calculated with unweighted UniFrac matrix. Results were significantly different (P = 0.001) at T1 and T2. (**b**) Relative abundance of bacteria phyla, expressed in percentage. AB and *L. rhamnosus* JB-1 treatments were started 1 week before pups' delivery and continued up until weaning. n = 4-5 CT, 4 AB, 3 AB/JB1.Tx: Treatment. CT: control; AB: antibiotic; AB/JB1: antibiotic and *L. rhamnosus* JB-1.

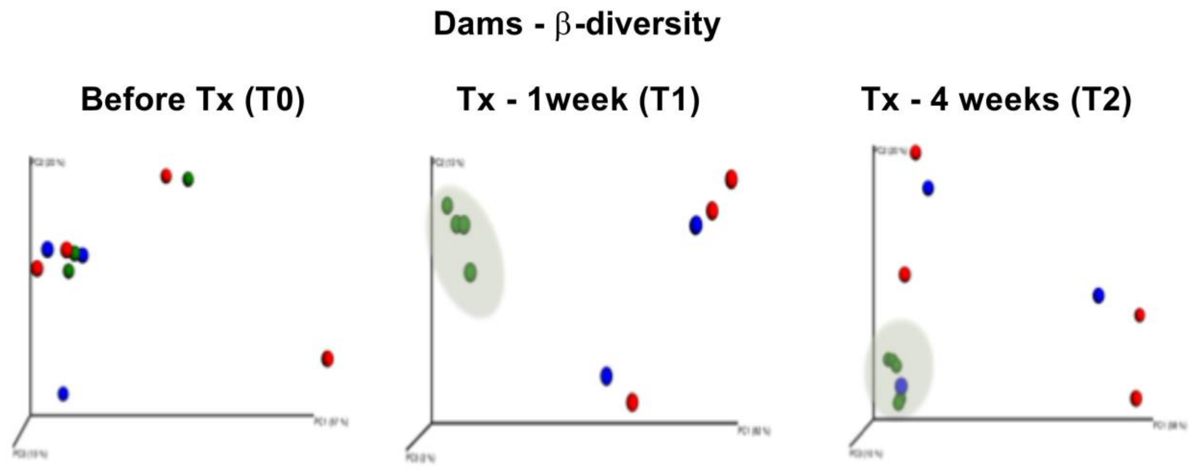

Supplementary Fig. 6: **Microbial communities of dams at three different time points using the weighted UniFrac matrix**

Before AB treatment (T0). One week after AB treatment (day of pups delivery, T1) *P* =0.001. Four weeks after AB treatment (day of pups weaning, T2) *P* =0.001. CT: control (in green); AB: antibiotic (in red); AB/JB1: antibiotic and *L. rhamnosus* JB-1 (in blue).

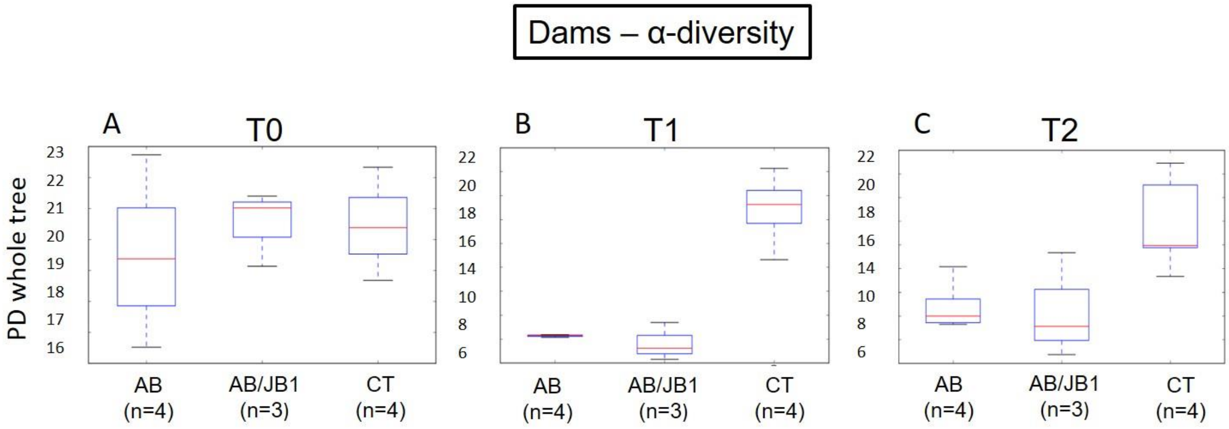

Supplementary Fig. 7: **Boxplots for community richness (α-diversity) in dams at three different time points**

(**a**) before AB treatment (T0); (**b**) one week after AB treatment (day of delivery, T1); (**c)** four weeks after AB treatment (day of weaning). Data shown are Faith's phylogenetic diversity (PD). CT: control; AB: antibiotic; AB/JB1: antibiotic and *L. rhamnosus* JB-1.

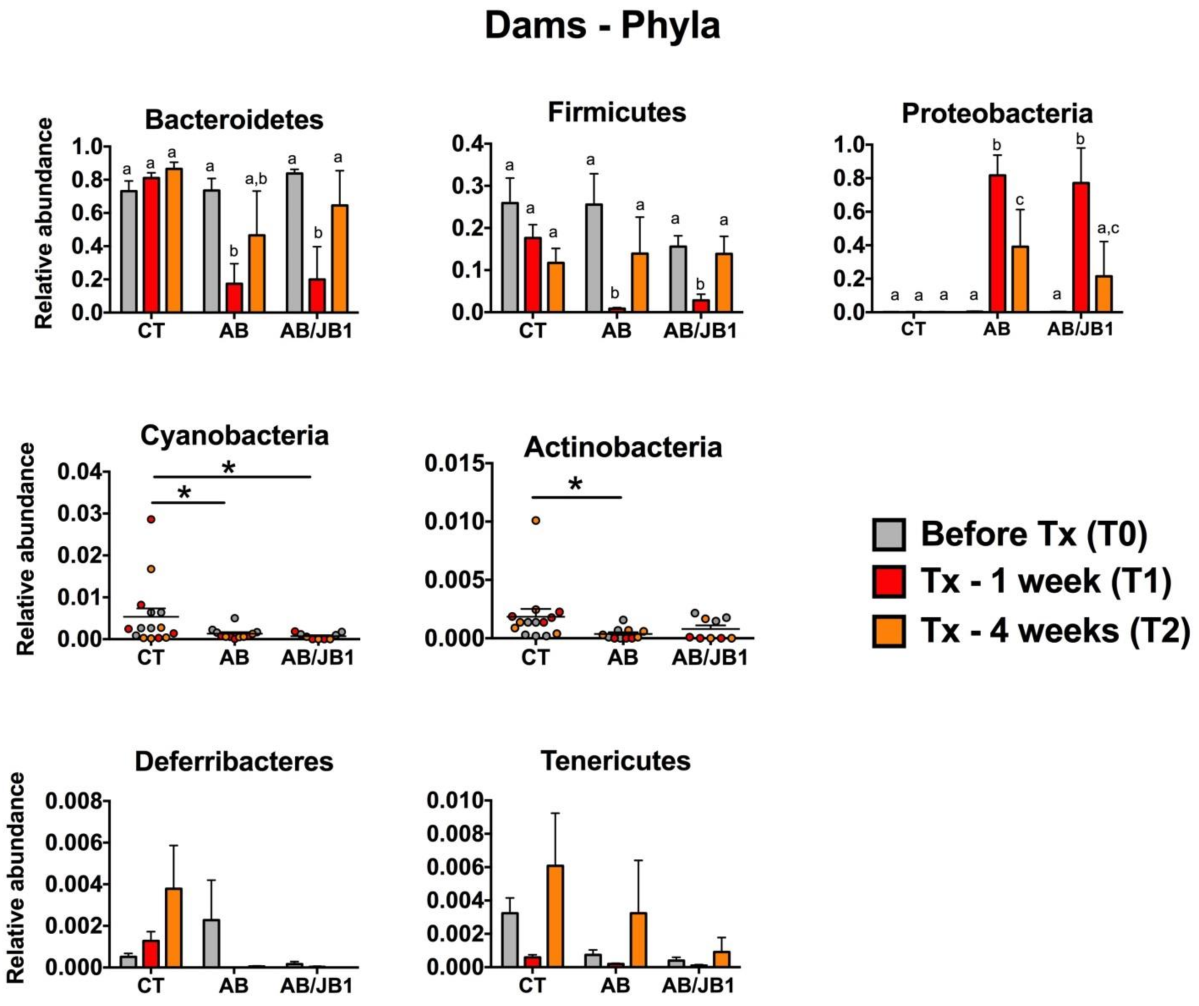

Supplementary Fig. 8: **Relative abundance of bacterial phyla in dams**

Mixed factorial ANOVA was performed to detect a main effect of treatment (before Tx (T0) and after Tx (T1 – T2)), a main effect of group (CT, AB, AB/JB1) and an interaction treatment*group. Significant main effects of treatment and group were observed for Bacteroidetes, Firmicutes and Proteobacteria, while significant interactions were observed for Bacteroidetes and Proteobacteria but not for Firmicutes. For the phyla Cyanobacteria and Actinobacteria, only a significant main effect of group was detected, while no significant main effect of treatment and no significant interaction were observed. No significant main effect of

treatment and group and no interaction were observed for Deferribacteres and Tenericutes. Results are means ± SEM (n = 4 CT, 4 AB, 3 AB/JB1). Different superscript letters indicate statistically significant difference, $P < 0.05$, after Bonferroni correction for multiple comparisons. Tx: treatment; CT: control; AB: antibiotic; AB/JB1: antibiotic and *L. rhamnosus* JB-1.

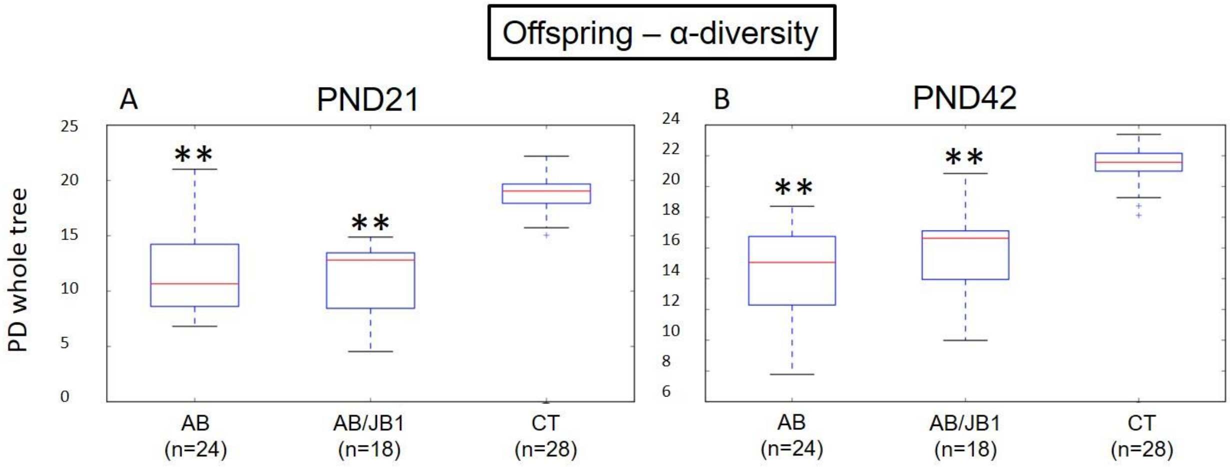

Supplementary Fig. 9: **Boxplots for community richness (α-diversity) in pups**

Alpha-diversity was measured at two time points: (**a**) postnatal day 21 (PND21) (weaning) and (**b**) postnatal day 42 (PND42). Data shown are Faith's phylogenetic diversity (PD). ** $P < 0.01$ vs CT. CT: control; AB: antibiotic; AB/JB1: antibiotic and *L. rhamnosus* JB-1.

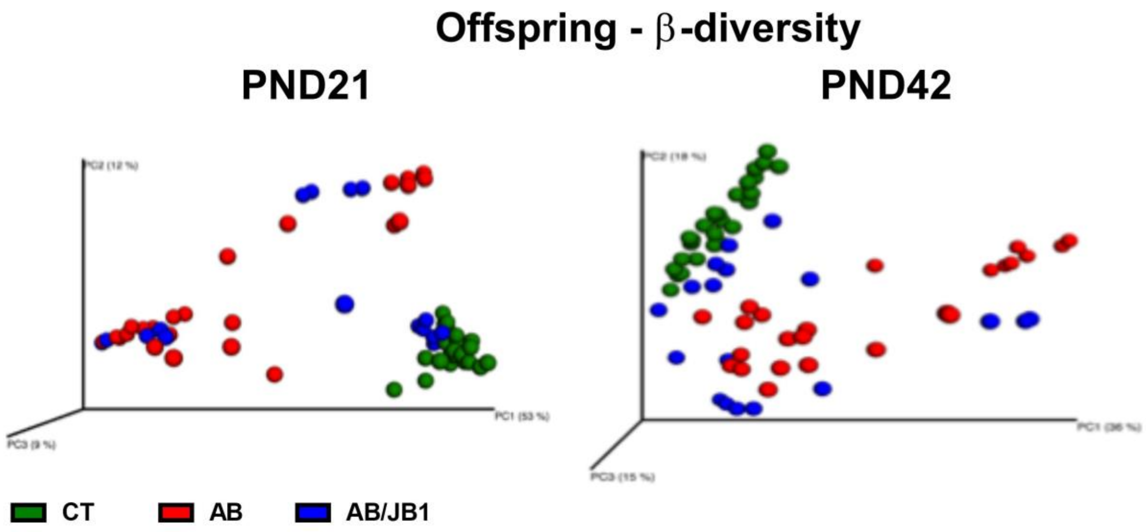

Supplementary Fig. 10: **Microbial communities of pups using the weighted UniFrac matrix**

Beta-diversity was measured at postnatal day 21 (PND21) (weaning), *P* = 0.001 and at postnatal day 42 (PND42), *P* = 0.001. (n = 28 CT, 24 AB and 18 AB/JB1). CT: control; AB: antibiotic; AB/JB1: antibiotic and *L. rhamnosus* JB-1.

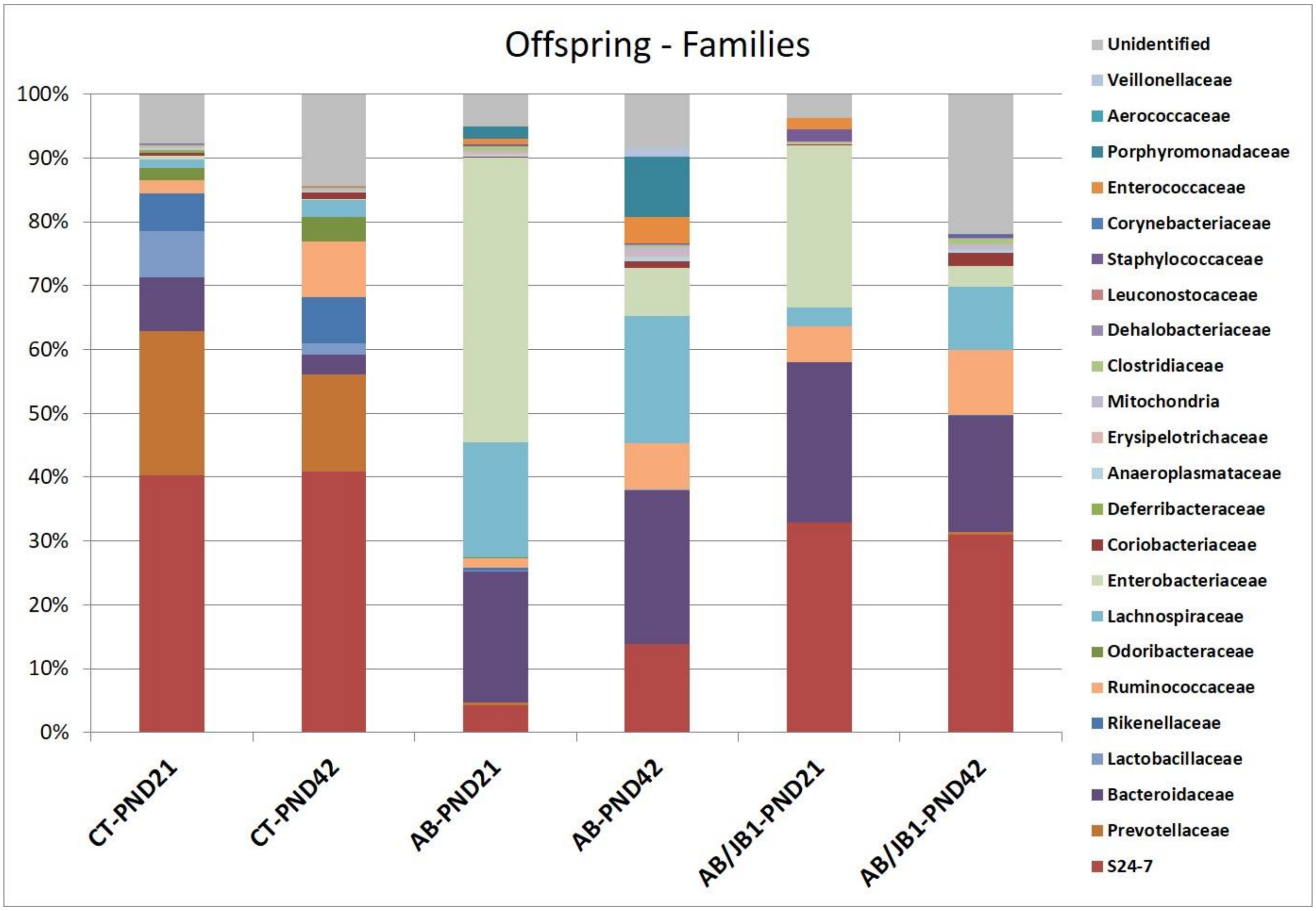

Supplementary Fig. 11: **Relative abundance (%) of bacterial families in offspring**

Relative abundance was measured at 3-weeks old (PND21) and 6-weeks old (PND42) (n = 28 CT, 24 AB and 18 AB/JB1). CT: control; AB: antibiotic; AB/JB1: antibiotic and *L. rhamnosus* JB-1.

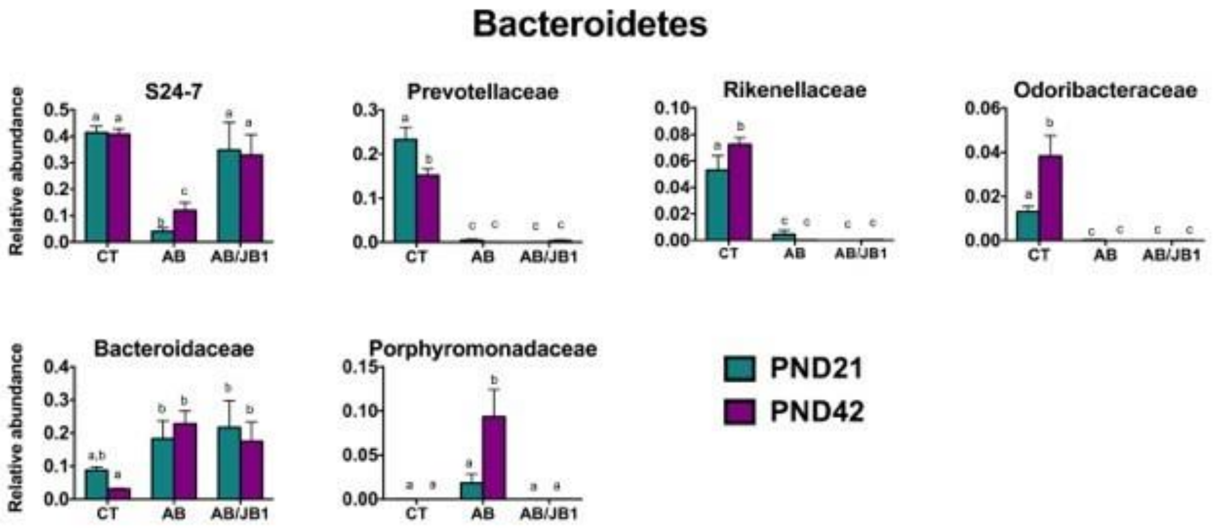

Supplementary Fig. 12: **Relative abundance of the bacterial families belonging to the phylum Bacteroidetes in offspring**

Factorial ANOVA was used to detect a main effect of time (PND21, PND42), a main effect of treatment (CT, AB, AB/JB1) and a significant interaction time*treatment. A significant main effect of treatment ($P < 0.05$) and a significant ($P < 0.05$) or marginally significant ($P < 0.10$) interaction time*treatment was found for all bacterial families. A significant ($P < 0.05$) main effect of time was found for the families Prevotellaceae, Odoribacteraceae and Porphyromonadaceae. Results are means ± SEM (n = 28 CT, 24 AB and 18 AB/JB1). Different superscript letters indicate statistically significant difference, $P < 0.05$ after Bonferroni correction for multiple comparisons. PND: postnatal day; CT: control; AB: antibiotic; AB/JB1: antibiotic and *L. rhamnosus* JB-1.

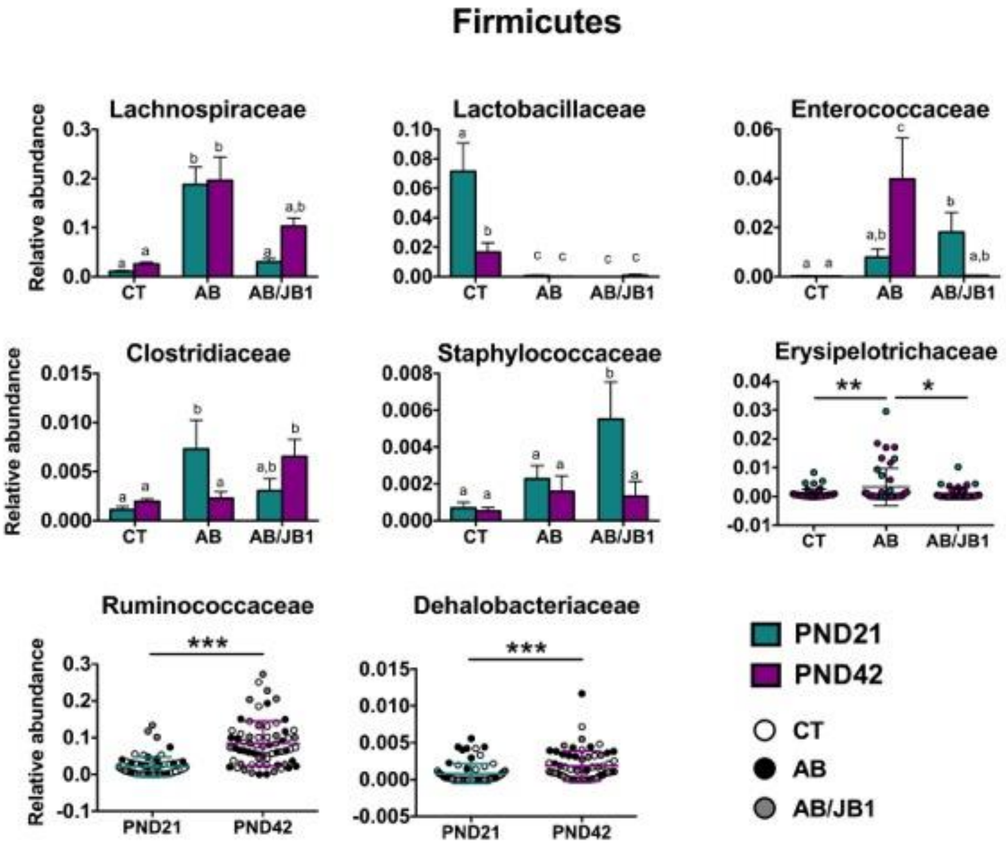

Supplementary Fig. 13: **Relative abundance of bacterial families belonging to the phylum Firmicutes in offspring**

Factorial ANOVA was used to detect the main effect of time (PND21, PND42), the main effect of treatment (CT, AB, AB/JB1) and the interaction treatment*time. For the family Erysipelotrichaceae, only a main effect of treatment was detected, while no main effect of time and no interaction were observed. For the families Ruminoccoccaceae and Dehalobacteriaceae, only a main effect of time was detected while no main effect of treatment or interaction were observed. Results are means ± SEM (SD for Erysipelotrichaceae, Ruminoccoccaceae and Dehalobacteriaceae)(n = 28 CT, 24 AB and 18 AB/JB1). Different superscript letters indicate statistically significant difference, $P < 0.05$ after Bonferroni correction for multiple comparisons. PND: postnatal day; CT: control; AB: antibiotic; AB/JB1: antibiotic and *L. rhamnosus* JB-1.

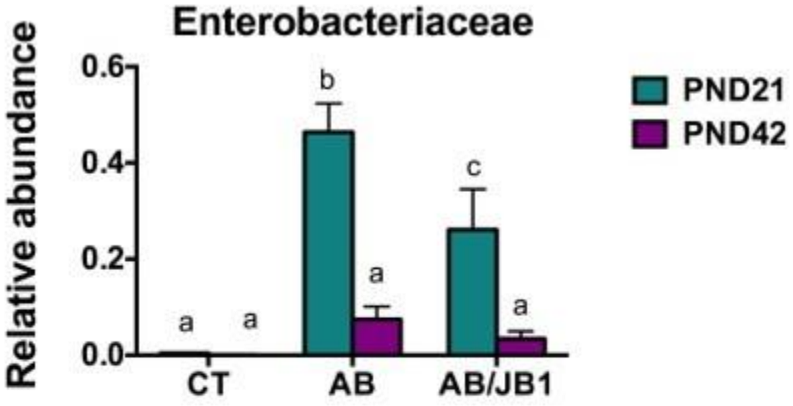

Supplementary Fig. 14: **Relative abundance of the family Enterobacteriaceae in offspring at 3- and 6-weeks old (PND21 and 42)**

Factorial ANOVA revealed a main effect of treatment ($F_{2,63} = 24.13$, $P < 0.001$), a main effect of time ($F_{1,63} = 45.69$, $P < 0.001$) and a significant interaction treatment*time ($F_{2,63} = 15.44$, $P < 0.001$). Post-hoc tests were then used to compare groups with Bonferroni adjustment for multiple comparisons. Results are means ± SEM (n = 28 CT, 24 AB and 18 AB/JB1). Different superscript letters indicate statistically significant difference, $P < 0.05$. PND: postnatal day; CT: control; AB: antibiotic; AB/JB1: antibiotic and *L. rhamnosus* JB-1.

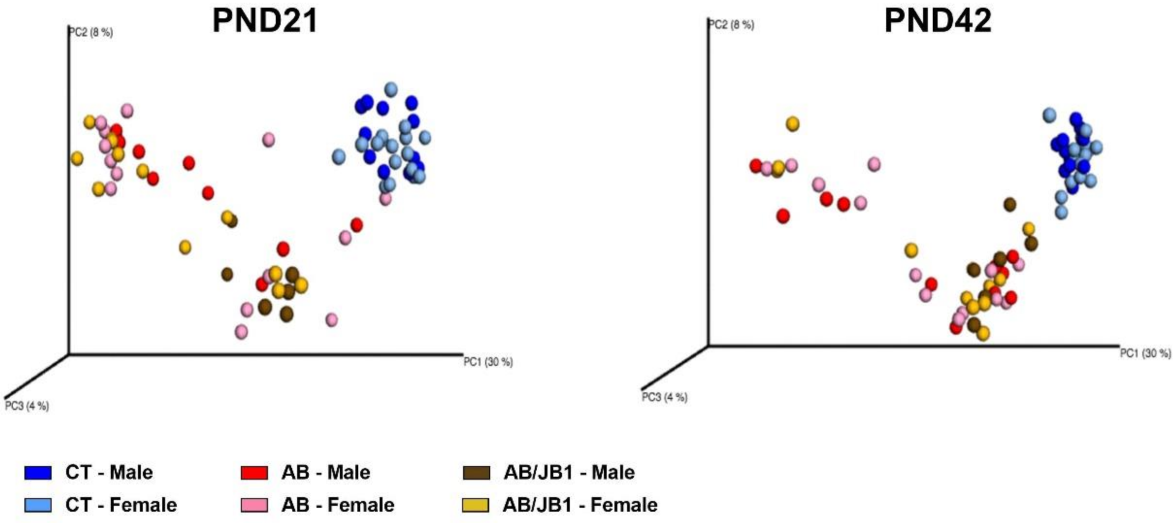

Supplementary Fig. 15: **Microbial communities of pups at PND21 and PND42**.

β-diversity was calculated using unweighted Unifrac matrix showing no difference between males and females (n = 11M/17F CT, 11M/13F AB and 6M/12F AB/JB1). PND: postnatal day; CT: control; AB: antibiotic; AB/JB1: antibiotic and *L. rhamnosus* JB-1.

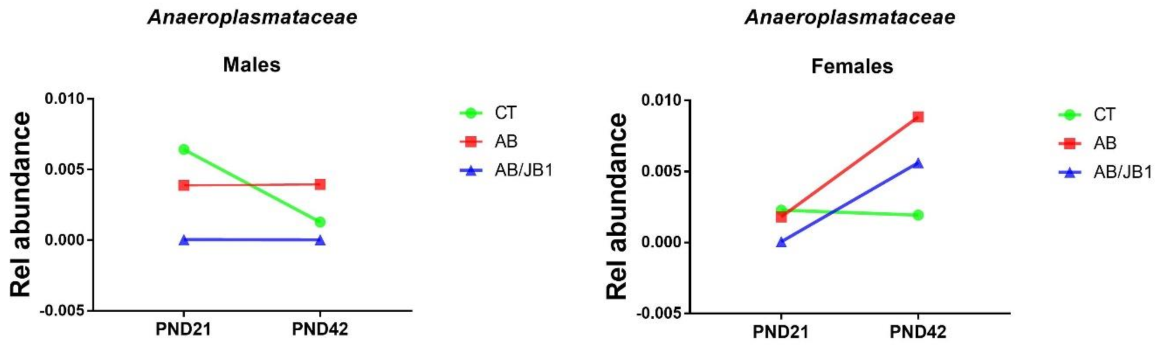

Supplementary Fig. 16: **Relative abundance of Anaeroplasmataceae (phylum Tenericutes/ class Mollicutes) at PND21 and PND42 in males and females**

Analysis revealed significant time* treatment ($F_{(2,60)}$ = 5.2, P = 0.009) and time*sex interaction ($F_{(1,60)}$ = 10.52, P = 0.002) suggesting that the change of the level of this bacteria from weaning to 6-week old is different between treatment groups as well as between male and female mice (n = 11M/17F CT, 11M/12F AB and 6M/11F AB/JB1). PND: postnatal day; CT: control; AB: antibiotic; AB/JB1: antibiotic and *L. rhamnosus* JB-1; M: male; F: female.

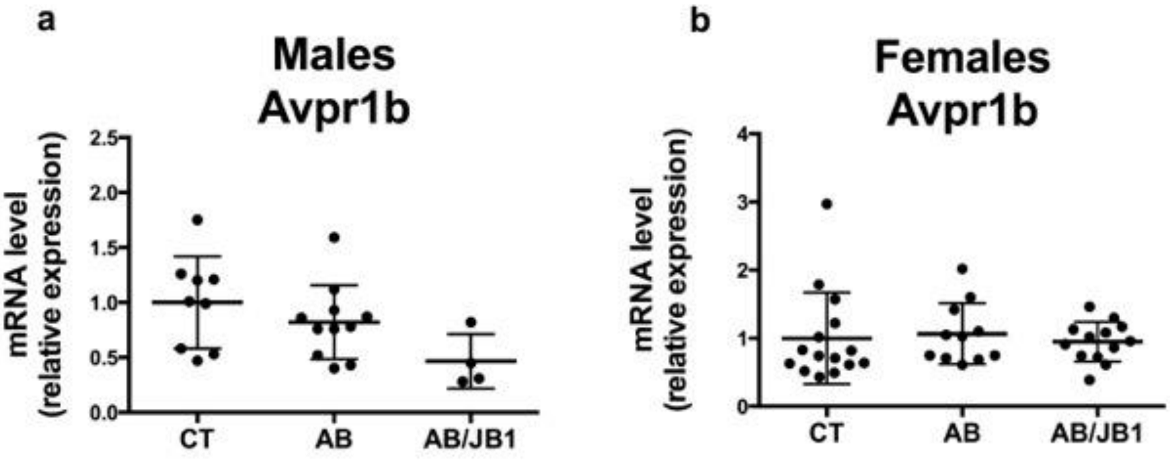

Supplementary Fig. 17: **Hippocampal expression of arginine vasopressin receptor 1B (Avpr1b)**

mRNA expression measured in the hippocampus of (**a**) males (n = 24) and (**b**) females (n = 39). No significant difference was observed by using one-way ANOVA ($P > 0.05$). Results are means ± SD. CT: control; AB: antibiotic; AB/JB1: antibiotic and *L. rhamnosus* JB-1.

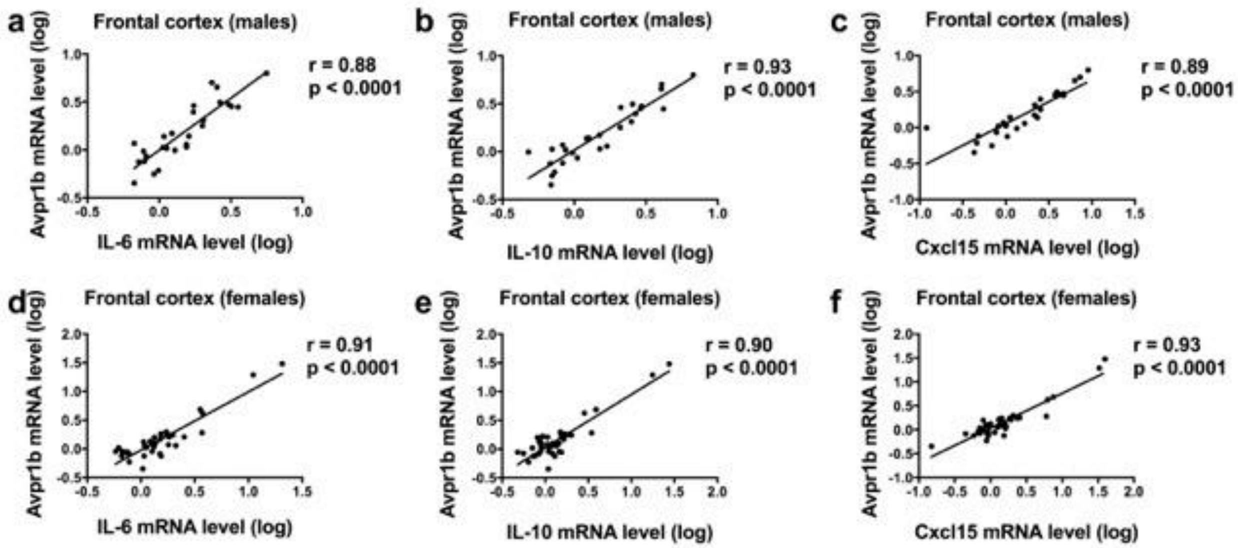

Supplementary Fig. 18: **Correlations between expression of cytokines and Avpr1b measured in the frontal cortex of both males and females**.

r represents Pearson correlation coefficient with corresponding p-value, n = 27 males and 41 females.

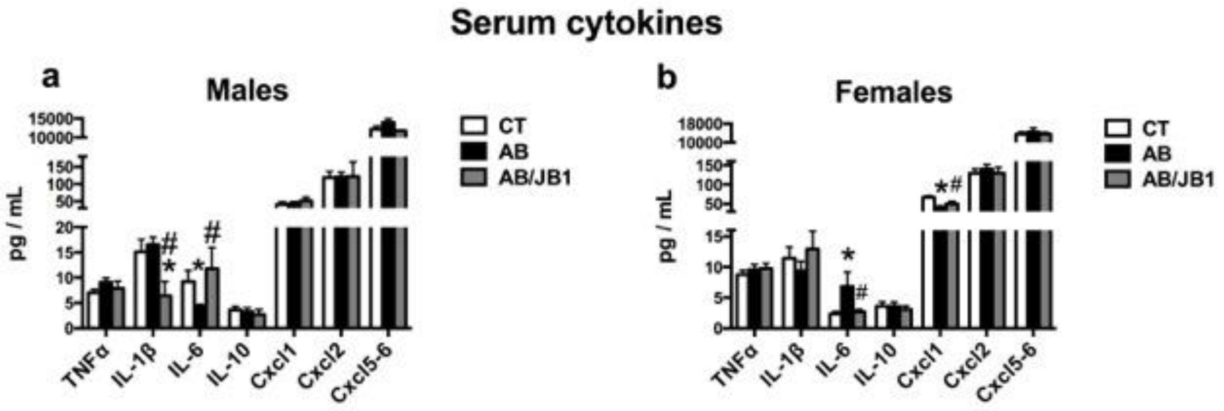

Supplementary Fig. 19: **Serum cytokines**

Inflammatory cytokines (TNFα, IL-1β, IL-6, IL-10) and chemokines functional IL-8 homologues (Cxcl1/KC, Cxcl2/MIP-2a, Cxcl5-6/LIX) were measured in males (**A**) and females (**B**). Results are means ± SEM (n = 28 males, 42 females) (one-way ANOVA). Different superscript letters indicate statistically significant difference, $P < 0.05$. * $P < 0.05$ vs CT. # $P < 0.05$ vs AB. CT: control; AB: antibiotic; AB/JB1: antibiotic and *L. rhamnosus* JB-1.

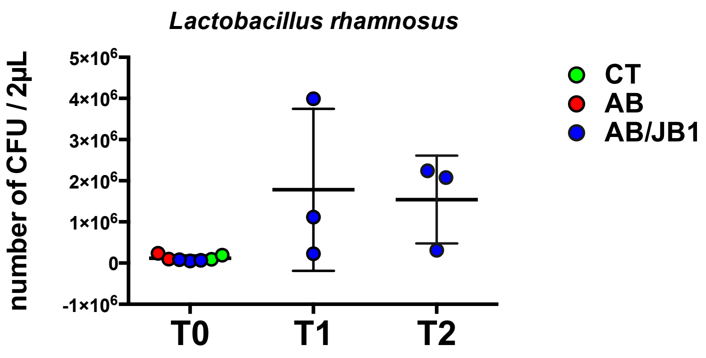

T0 = before Tx
T1 = after 1 week of Tx
T2 = after 4 weeks of Tx

Supplementary Fig. 20: **Quantification of *Lactobacillus rhamnosus* in the feces of dams**

L. rhamnosus was low but detectable in all groups before the treatment, then detectable, at T1 and T2, only in dams that receive the supplementation with *L. rhamnosus* JB-1. Tx: treatment; CT: control; AB: antibiotic; AB/JB1: antibiotic and *L. rhamnosus* JB-1.

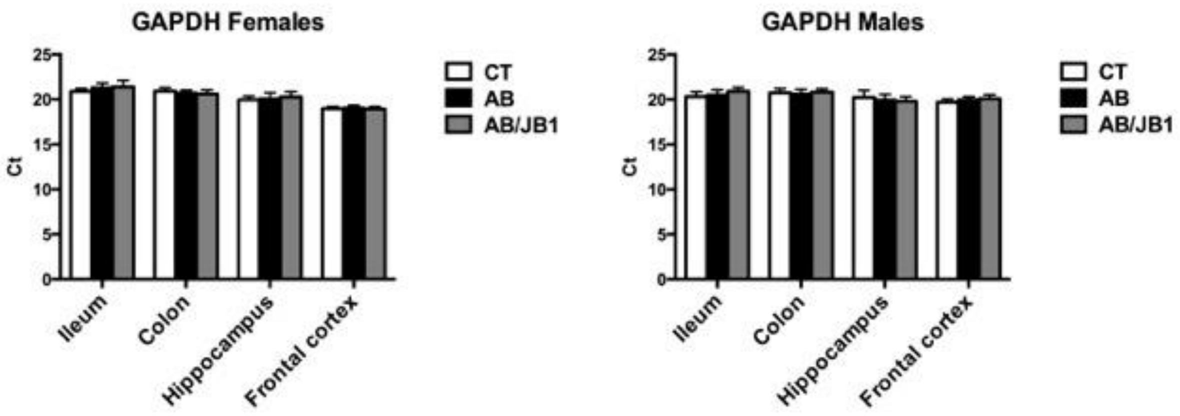

Supplementary Fig. 21: **mRNA expression of the housekeeping gene GAPDH measured in different tissues in male and female mice**

The experimental treatments did not affect GAPDH expression. CT: control; AB: antibiotic; AB/JB1: antibiotic and *L. rhamnosus* JB-1.

Males hippocampus (membrane 1)

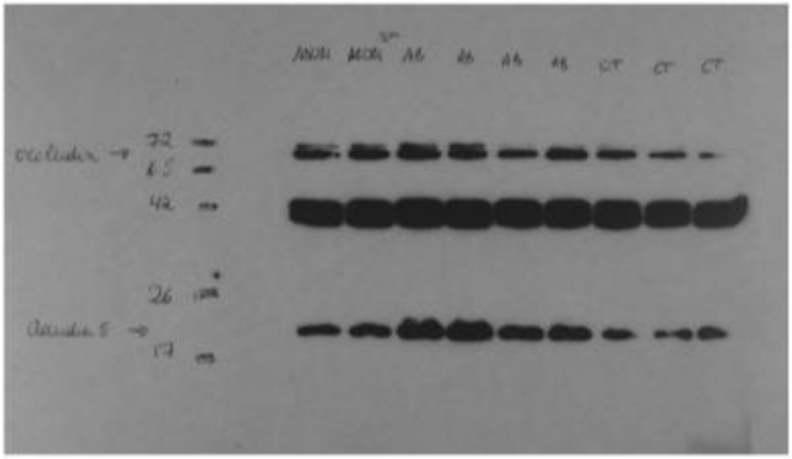

Males hippocampus (membrane 2)

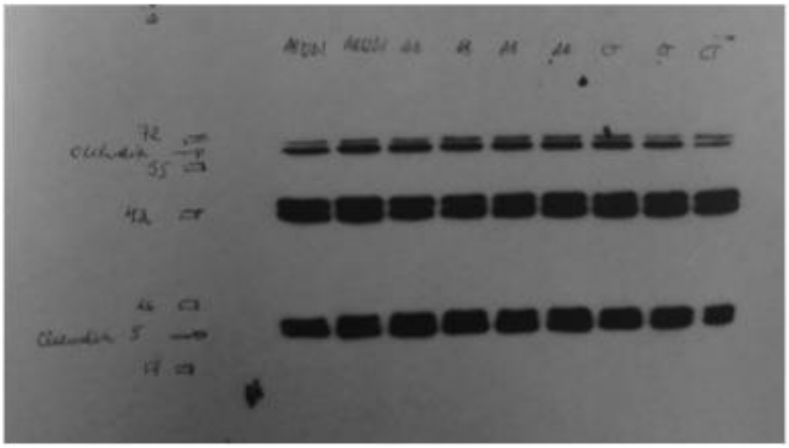

Supplementary Fig. 22: **Full blots of tight junction protein expression in the hippocampus of male mice**

In total, protein extracts from 5 CT, 8 AB and 4 AB/JB1 have been measured. CT: control; AB: antibiotic; AB/JB1: antibiotic and *L. rhamnosus* JB-1.

Supplementary Table 1: Effect of early life antibiotic treatment of the relative abundance of family Lactobacillaceae and genus *Lactobacillus*

	CT		AB		AB/JB1	
Relative abundance (%)	PND21	PND42	PND21	PND42	PND21	PND42
Lactobacillaceae (family)	7.1 ± 9.9	1.7 ± 3.3	0.1 ± 0.2	0.01 ± 0.001	0.01 ± 0.001	0.1 ± 0.2
Lactobacillus (genus)	6.7 ± 0.08	1.6 ± 0.03	0.1 ± 0.004	0.0	0.0	0.1 ± 0.002

Results are means ± SD. CT: control; AB: antibiotic; AB/JB1: antibiotic and *L. rhamnosus* JB-1.

Supplementary Table 2: mRNA expression of tight junctions and inflammatory cytokines in the ileum of female mice

	CT	AB	AB/JB1
	Mean ± SD, n = 16-17	Mean ± SD, n = 13	Mean ± SD, n = 13
Occludin	1.0 ± 0.15	1.23 ± 0.41	1.07 ± 0.42
ZO-1	1.0 ± 0.26	0.91 ± 0.21	0.79 ± 0.19
TNFα	1.0 ± 0.33	0.86 ± 0.46	0.77 ± 0.42
IL-1β	1.0 ± 0.34	0.85 ± 0.26	0.85 ± 0.27
IL-6	1.0 ± 0.55	0.65 ± 0.27	0.86 ± 0.53
IL-10	1.0 ± 0.53	0.53 ± 0.25*	0.70 ± 0.29
Cxcl15	1.0 ± 0.45	1.73 ± 0.66*	1.21 ±0.74

. * $P < 0.05$ compared to CT (one-way ANOVA followed by Tukey's multiple comparison post-hoc tests). CT: control; AB: antibiotic; AB/JB1: antibiotic and *L. rhamnosus* JB-1.

Supplementary Table 3: mRNA expression of tight junctions and inflammatory cytokines in the ileum of male mice

	**CT**	**AB**	**AB/JB1**
	Mean ± SD, n = 10-11	Mean ± SD, n = 11-12	Mean ± SD, n = 5
Occludin	1.0 ± 0.21	1.10 ± 0.27	0.90 ± 0.36
ZO-1	1.0 ± 0.23	1.25 ± 0.30	1.06 ± 0.32
TNFα	1.0 ± 0.22	0.65 ± 0.19*	0.97 ± 0.16
IL-1β	1.0 ± 0.24	0.77 ± 0.18*	0.80 ± 0.10
IL-6	1.0 ± 0.42	1.02 ± 0.48	0.86 ± 0.33
IL-10	1.0 ± 0.39	0.43 ± 0.23*	0.67 ± 0.40
Cxcl15	1.0 ± 0.38	0.79 ± 0.28	0.89 ± 0.62

* $P < 0.05$ compared to CT (one-way ANOVA followed by Tukey's multiple comparison post-hoc tests). CT: control; AB: antibiotic; AB/JB1: antibiotic and *L. rhamnosus* JB-1.

Supplementary Table 4: Correlations between mRNA expression of tight junctions and inflammatory cytokines measured in the hippocampus of male mice

Hippocampus		IL-6	IL-10	Cxcl15
Occludin	Spearman's rho	-0.404	-0.361	-0.357
	P-value	0.049	0.084	0.086
	N	24	24	24
Cldn5	Spearman's rho	-0.406	-0.342	-0.346
	P-value	0.049	0.09	0.098
	N	24	24	24

Correlations were calculated with Spearman's coefficient.

Supplementary Table 5: Correlations between mRNA expression of tight junctions and inflammatory cytokines measured in the frontal cortex of male and female mice

Frontal cortex		IL-6	IL-10	Cxcl15
Occludin (males)	Spearman's rho	0.716	0.718	0.666
	P-value	< 0.001	< 0.001	< 0.001
	N	28	28	28
Cldn5 (females)	Spearman's rho	0.473	0.462	0.465
	P-value	< 0.001	< 0.001	< 0.001
	N	42	42	42

Correlations were calculated with Spearman coefficient.

Supplementary Table 6: **Primer sequences used for real time quantitative PCR**

Gene	Access Number	Forward Primer (5' to 3')	Reverse Primer (5' to 3')
Avpr1b	NM_011924	TCTACTCTCCGTCTTAGCCTTAACCT	CTCCATCCACCTGCTCCAA
Claudin-5 (Cldn5)	NM_013805	TCAGCTTCCCGGTCAAGTACTC	CCGCCCTTAGACATAGTTCTTCTT
Cxcl15 (IL-8)	NM_011339	CAGGCCACAGACGGACATG	GGACGAAGATGCCTAGGTTAAGG
GAPDH	NM_001289726	AGCTTGTCATCAACGGGAAG	TTTGATGTTAGTGGGGTCTCG
IL-1β	NM_008361	TCGCTCAGGGTCACAAGAAA	CATCAGAGGCAAGGAGGAAAAC
IL-6	NM_031168	CTGCAAGAGACTTCCATCCAGTT	GAAGTAGGGAAGGCCGTGG
IL-10	NM_010548	GCTCTTACTGACTGGCATGAG	CGCAGCTCTAGGAGCATGTG
Occludin	NM_008756	TGAACAGCCCCCCAATGT	TCAACTCTTTCCGCATAGTCAGAT
TNFα	NM_013693	CCACCACGCTCTTCTGTCTAC	TGGGCTACAGGCTTGTCACT
ZO-1 (Tjp1)	NM_001163574	GTGGATAGATCATTCAGTGAGAAACGT	TGGGCGCCCTTGGAA
